# Dye Sensitization for Ultraviolet Upconversion Enhancement

**DOI:** 10.3390/nano11113114

**Published:** 2021-11-18

**Authors:** Mingkai Wang, Hanlin Wei, Shuai Wang, Chuanyu Hu, Qianqian Su

**Affiliations:** 1 Institute of Nanochemistry and Nanobiology, Shanghai University, Shanghai 200444, China; wmk19961108@163.com (M.W.); hanliwei@cityu.edu.hk (H.W.); m18937353231@163.com (S.W.); 2 Department of Stomatology, Tongji Hospital, Tongji Medical College, Huazhong University of Science and Technology, Wuhan 430030, China; 3 School of Stomatology, Tongji Medical College, Huazhong University of Science and Technology, Wuhan 430030, China; 4 Hubei Province Key Laboratory of Oral and Maxillofacial Development and Regeneration, Wuhan 430022, China

**Keywords:** lanthanide nanoparticles, ultraviolet upconversion, dye sensitization, heterogeneous nanoparticles, energy transfer, luminescence enhancement

## Abstract

Upconversion nanocrystals that converted near-infrared radiation into emission in the ultraviolet spectral region offer many exciting opportunities for drug release, photocatalysis, photodynamic therapy, and solid-state lasing. However, a key challenge is the development of lanthanide-doped nanocrystals with efficient ultraviolet emission, due to low conversion efficiency. Here, we develop a dye-sensitized, heterogeneous core–multishelled lanthanide nanoparticle for ultraviolet upconversion enhancement. We systematically study the main influencing factors on ultraviolet upconversion emission, including dye concentration, excitation wavelength, and dye-sensitizer distance. Interestingly, our experimental results demonstrate a largely promoted multiphoton upconversion. The underlying mechanism and detailed energy transfer pathway are illustrated. These findings offer insights into future developments of highly ultraviolet-emissive nanohybrids and provide more opportunities for applications in photo-catalysis, biomedicine, and environmental science.

## 1. Introduction

Lanthanide-doped upconversion nanoparticles can absorb near-infrared (NIR) laser light and emit visible and ultraviolet light, with potential applications in bioimaging [1,2,3,4,5], biotherapy [6,7,8,9,10,11,12], and so on. In particular, the applications of these nanoparticles in optogenetic [13,14], photothermal [15,16], and photodynamic [17,18,19] therapy could be achieved via ultraviolet (UV) light emission under NIR excitation. Although UV light can be obtained by Nd^3+^- and Yb^3+^-sensitized upconversion [17,18,20,21], it is challenging to realize the high luminescence intensity needed to satisfy the minimum requirement of biological applications. This obstacle can be addressed in several ways: by controlling dopant composition [22], nanoparticle phase and size [23], excitation beam pulse width [24], and nanoparticle core–shell design [21,25,26,27,28,29]. Very recently, our group has made significant progress in overcoming the difficulty using an upconverted excitation lock-in (UCEL) strategy [30].

Hybrid systems are composed of inorganic nanoparticles and an organic dye, which can significantly strengthen the absorbance and expand the absorbance spectra of inorganic nanoparticles [31], leading to enhancement of their emission intensities. It has been demonstrated that NIR dye can effectively enhance the upconversion emission of lanthanide-doped nanoparticles [14,32,33,34]. However, previous studies have mainly focused on the analysis of visible upconversion emission. Little effort has been made to develop a hybrid nanoparticle with enhanced UV luminescence.

**Scheme 1:**
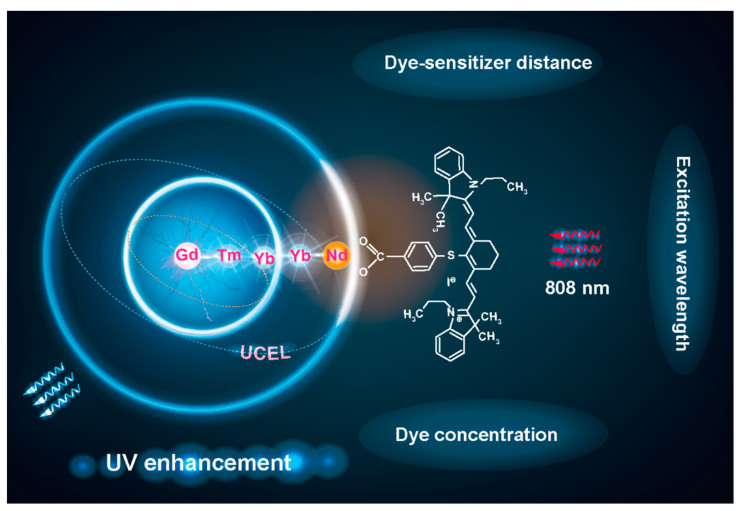
Schematic illustration of the key factors that influence UV enhancement in IR-806-loaded upconversion nanoparticles, including dye concentration, excitation wavelength, and dye–sensitizer distance.

In this study, we developed IR-806-loaded upconversion nanoparticles (Gd-CS_Y_S_2_S_3_@IR-806) with enhanced upconversion emission in the UV spectral region. The key factors that influence upconversion emission were studied, including dye concentration, excitation wavelength, and distance between the dye and the sensitizer Nd^3+^ (Scheme 1). We also demonstrated the dominant effect of ligand loading on multiphoton upconversion. In addition, the upconversion mechanism and the energy transfer pathway in Gd-CS_Y_S_2_S_3_@IR-806 hybrid nanoparticles were carefully studied. This study provides new insights into the mechanistic understanding of UV upconversion luminescence in hybrid nanoparticles and enables new opportunities for these nanomaterials in a broad range of applications.

## 2. Materials and Methods

### 2.1. Materials

Gd(CH_3_CO_2_)_3_·xH_2_O (99.9%), Nd(CH_3_CO_2_)_3_·xH_2_O (99.9%), Y(CH_3_CO_2_)_3_·xH_2_O (99.9%), Yb(CH_3_CO_2_)_3_·xH_2_O (99.9%), Tm(CH_3_CO_2_)_3_·xH_2_O (99.9%), NaOH (>98%), NH_4_F (>98%), chloroform(99.9%), oleic acid (OA, 90%), and 1-octadecene (ODE, 90%) were all purchased from Sigma-Aldrich (Shanghai, China). IR-806 is supported by Dr. Sanyang Han from the University of Cambridge. IR-780 iodide, 4-mercaptobenzoic acid, and N,N-dimethylformamide (DMF, anhydrous, 99.8%), as raw materials of IR-806, were obtained from Sigma-Aldrich (London, UK). Dichloromethane (DCM, AR) and diethyl ether (AR), as solvents for the synthesis of IR-806, were obtained from Lab-Scan (London, United Kingdom). Chloroform (AR), cyclohexane (AR), and ethanol (AR) were purchased from Sinopharm Chemical Reagent Co., Ltd. (Shanghai, China). All chemicals and reagents were used as received without further purification unless otherwise noted.

### 2.2. Characterization

Luminescence emission measurements were obtained with an FS5 (Edinburgh, UK) conjugated with 808 nm (CNI, MDL-III-808-2.5W, China), 793 nm (CNI, MDL-III-793-2.0W, China), and 980 nm (CNI, MDLIII-980-2.0W, China) diode lasers at room temperature. The decay curves were recorded by a lifetime spectrometer (FS5, Edinburgh, UK), in conjunction with pulsed 808 nm, 793 nm, and 980 nm diode lasers and a picosecond pulsed light emitting diode (EPLED-270). Low-resolution transmission electron microscopy (TEM) measurements were carried out on an HT7700 field emission transmission electron microscope operated at an acceleration voltage of 120 kV. The energy-dispersive X-ray (EDX) spectrum was obtained with an HT7700 field emission transmission electron microscope equipped with an Oxford Instruments system. High-resolution TEM images were obtained using an FEI Talos F200S transmission electron microscope operated at an acceleration voltage of 200 kV. HAADF-STEM and elemental mapping images were obtained using an FEI Talos F200X transmission electron microscope. Powder X-ray diffraction (XRD) analysis was performed on a Rigaku D/Max-2200 system equipped with a rotating anode and a Cu Kα radiation source (λ = 0.15418 nm). The excitation power density was measured using a TS5 laser power densitometer (Changchun New Industries Optoelectronics Technology, China). UV–vis absorption spectra were obtained using a PerkinElmer LAMBDA 750 ultraviolet–visible–near-infrared spectrometer and a Hitachi U-3010 spectrophotometer. All spectra were recorded under identical experimental conditions unless otherwise noted. Key experiments were repeated three times, and all other experiments were repeated twice.

### 2.3. Method

#### 2.3.1. Synthesis of NaGdF_4_:49%Yb,1%Tm Core Nanocrystals

NaGdF_4_ doped with 49 mol % of Yb and 1 mol % of Tm (NaGdF_4_:49%Yb,1%Tm) was synthesized via a modified literature procedure [28,35,36]. A water solution of Gd(CH_3_CO_2_)_3_ (0.067 g; 0.2 mmol), Yb(CH_3_CO_2_)_3_ (0.069 g; 0.196 mmol), and Tm(CH_3_CO_2_)_3_ (0.001 g; 0.004 mmol) was combined with OA (5 mL) and ODE (5 mL) in a 50 mL two-neck round-bottom flask. The mixture was heated to 150 °C and maintained at this temperature for 1.5 h to form the lanthanide–oleate precursor. After cooling to 50 °C, a methanol solution consisting of NH_4_F (0.05 g; 1.36 mmol) and NaOH (0.04 g; 1 mmol) was added to the mixture and stirred for 30 min. The solution was heated to 100 °C for 20 min in vacuo to remove methanol. The resulting solution was quickly heated to 300 °C and maintained at this temperature for 1.5 h with nitrogen before cooling to room temperature. The obtained nanocrystals were precipitated by centrifugation at 8000 rpm for 5 min and then washed with cyclohexane and ethanol three times. The core nanoparticles were dispersed in cyclohexane (4 mL) for further shell coating.

#### 2.3.2. Synthesis of NaGdF_4_:49%Yb,1%Tm@NaYF_4_:20%Yb Core–Shell Nanocrystals

The synthesis procedure for core–shell nanoparticles was similar to that in our previous paper [36]. We use the obtained NaGdF_4_:49%Yb,1%Tm nanocrystals as seeds for subsequent shell coating. NaYF_4_ with 20 mol % of Yb (NaYF_4_:20%Yb) precursor was prepared via the same procedure as mentioned above, except that different amounts of OA (3 mL) and ODE (7 mL) were used. After cooling to 80 °C, the cyclohexane solution of NaGdF_4_:Yb/Tm nanoparticles (4 mL) was added and kept at 80 °C for 30 min to remove cyclohexane. Then, a methanol solution of NH_4_F (0.05 g; 1.36 mmol) and NaOH (0.04 g; 1 mmol) was added to the mixture and stirred at 50 °C for 30 min. Subsequently, the mixture was heated to 100 °C for 20 min in vacuo to remove methanol. The solution was then heated to 300 °C for 1.5 h under a nitrogen atmosphere. After cooling to room temperature, the core–shell nanoparticles were collected and washed using the same post-treatment approach as for core nanocrystals. NaGdF_4_@NaGdF_4_:49%Yb,1%Tm and NaYF_4_@NaGdF_4_:49%Yb,1%Tm were synthesized using a similar method to core–shell nanocrystals except for the use of NaGdF_4_ and NaYF_4_ as seeds.

#### 2.3.3. Synthesis of NaGdF_4_:49%Yb,1%Tm@NaYF_4_:20%Yb@NaGdF_4_:50%Nd,10%Yb and NaGdF_4_:49%Yb,1%Tm@NaYF_4_:20%Yb@NaGdF_4_:50%Nd,10%Yb@NaGdF_4_ Core–Multishell Nanocrystals

The following multishelled core–shell nanoparticles were prepared using a procedure similar to the NaGdF_4_:49%Yb,1%Tm@NaYF_4_:20%Yb core–shell nanoparticles: NaGdF_4_@NaGdF_4_:49%Yb,1%Tm@ NaYF_4_:20%Yb; NaYF_4_@NaGdF_4_:49%Yb,1%Tm@NaYF_4_: 20%Yb; NaGdF_4_@NaGdF_4_:49%Yb, 1%Tm@NaYF_4_:20%Yb@NaGdF_4_:50%Nd,10%Yb; NaYF_4_ @NaGdF_4_:49%Yb,1%Tm@NaYF_4_:20%Yb@NaGdF_4_:50%Nd,10%Yb;NaGdF_4_@NaGdF_4_:49%Yb,1%Tm@NaYF_4_:20%Yb@NaGdF_4_:50%Nd,10%Yb@NaGdF_4_; NaYF_4_@NaGdF_4_:49%Yb,1% Tm@NaYF_4_:20%Yb@ NaGdF_4_:50%Nd,10%Yb@NaGdF_4_.

#### 2.3.4. Preparation of Dye-Sensitized Upconversion Nanoparticles

The synthesis of IR-806 followed a well-established method [32]. Then, the IR-806 was dissolved in CHCl_3_ (0.01 mg/mL). The as-prepared core–multishell nanocrystals were centrifuged and dissolved in CHCl_3_ to a final concentration of 0.375 mg/mL. The samples were prepared by adding different amounts of IR-806 to Gd-CS_Y_S_2_S_3_ CHCl_3_ solution (4 mL) and stirring for 2 h at a speed of 700 rpm at room temperature before UV–vis–NIR absorption and standard fluorescence measurements. All samples were prepared and measured in a dark environment.

## 3. Results

### 3.1. Synthesis of Core–Multishell Upconversion Nanoparticles

**Figure 1:**
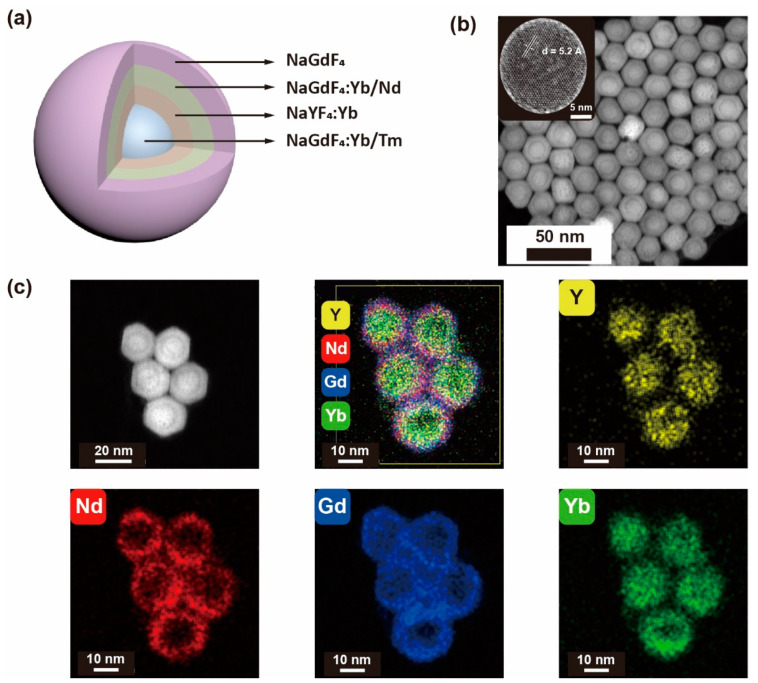
Schematic illustration and characterization of Gd-CS_Y_S_2_S_3_ heterogeneous nanoparticles. (**a**) Diagrammatic representation of Gd-CS_Y_S_2_S_3_ nanostructure. (**b**) High-angle annular dark-field scanning transmission electron microscopy (HAADF-STEM) image of Gd-CS_Y_S_2_S_3_ nanoparticles. Inset: high-resolution TEM of as-prepared Gd-CS_Y_S_2_S_3_ nanoparticle. (**c**) HAADF-STEM image and elemental mapping image of Gd-CS_Y_S_2_S_3_ nanoparticles, revealing the spatial distribution of the Y, Nd, Gd, and Yb elements in the heterogeneous nanoparticles.

We previously designed a heterogeneous core–multishell nanoparticle with enhanced UV upconversion emission, involving six- and five-photon upconversion processes [30]. The optimum doping concentration and nanoparticle design were determined according to our previous reports [36]. From our previous photoluminescence results, the optimized nanostructure was determined to be NaGdF_4_:49%Yb/1%Tm@NaGdF_4_:20%Yb@ NaGdF_4_:10%Yb/50%Nd@NaGdF_4_. Recently, we found that when the NaGdF_4_:20%Yb was replaced with NaYF_4_:20%Yb, UV emission was significantly enhanced due to the effective suppression of energy consumption induced by interior energy traps. Herein, we chose this heterogeneous nanostructure as an experimental model to further enhance upconversion emission in the UV range. We first synthesized the core–multishell NaGdF_4_:49%Yb,1%Tm@NaYF_4_:20%Yb@NaGdF_4_:10%Yb,50%Nd@NaGdF_4_ (Gd-CS_Y_S_2_S_3_) nanoparticles using a layer epitaxial growth method. Transmission electron microscopy (TEM) images showed that the nanoparticles had a uniform size of about 28 nm and the thickness of each layer was ~2 nm (Figure S1). The as-prepared nanoparticles were identified as the hexagonal phase by X-ray powder diffraction (XRD, JCPDS file number 27-0699, Figure S2). In addition, the constitution of the heterogeneous core–multishell nanostructures was confirmed by high-angle annular dark-field scanning transmission electron microscopy (HAADF-STEM), elemental mapping images and energy dispersive X-ray (EDX) spectra (Figure 1b,c, Figures S3 and S4), where brighter regions correspond to heavier elements (Gd, Yb, and Nd) and lighter regions correspond to lighter elements (Y).

### 3.2. Remarkable UV Enhancement

**Figure 2:**
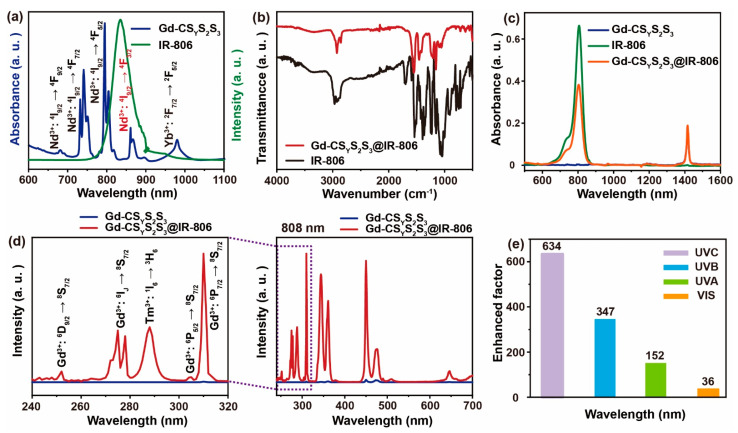
Preparation and characterization of Gd-CS_Y_S_2_S_3_@IR-806. (**a**) IR-806 emission spectrum and Gd-CS_Y_S_2_S_3_ nanoparticles absorption spectrum. (**b**) FTIR of Gd-CS_Y_S_2_S_3_@IR-806 and IR-806. (**c**) The absorption spectra of Gd-CS_Y_S_2_S_3_, IR-806, and Gd-CS_Y_S_2_S_3_@IR-806. (**d**) Emission spectra of Gd-CS_Y_S_2_S_3_ with and without IR-806 loading under 808 nm CW diode laser at a power density of 10 W/cm^2^. (**e**) The enhancement factors of upconversion emission were obtained by comparing the results for samples with and without IR-806 loading. The emission intensities were calculated by integrating the spectral intensities in the UVC (240–280 nm), UVB (280–320 nm), UVA (320–400 nm), and visible (400–650 nm) ranges.

To enhance upconversion emission in the UV range, we chose a near-infrared (NIR) fluorescent dye (IR-806) to sensitize upconversion nanoparticles, due to its intense absorption in the NIR range [33]. As shown in Figure 2a, the fluorescence spectrum of IR-806 has considerable overlap with the absorbance of Gd-CS_Y_S_2_S_3_ nanoparticles with Nd^3+^ (^4^F_3/2_→^4^I_9/2_) sensitizer, ensuring an effective energy transfer from IR-806 to the nanoparticles. We then utilized a modified Hummelen’s method to load the IR-806 onto the surface of Gd-CS_Y_S_2_S_3_ nanoparticles [32]. In addition, free IR-806 has an absorption band at 1708 cm^−1^ in the FTIR spectrum, corresponding to the stretching mode of –COOH. The absorption band at 1708 cm^−1^ disappeared when IR-806 was bound on to the surface of the nanoparticles. Nevertheless, absorption bands at 1560 and 1450 cm^−1^ were observed on Gd-CS_Y_S_2_S_3_@IR-806, corresponding to the antisymmetric and symmetric vibration modes of the –COO^−^ group. This indicates that the IR-806 carboxylic acid group was bound onto the surface of Gd-CS_Y_S_2_S_3_, since the carboxylic region changed [33]. The successful preparation of IR-806-loaded Gd-CS_Y_S_2_S_3_ nanoparticles was demonstrated by Fourier-transform infrared spectroscopy (FTIR) analysis (Figure 2b), which is consistent with the previous report [32]. In addition, we also compared the absorption spectra of Gd-CS_Y_S_2_S_3_, IR-806, and Gd-CS_Y_S_2_S_3_@IR-806. As shown in Figure 2c, Gd-CS_Y_S_2_S_3_@IR-806 nanoparticles showed an intense absorbance band peaking at ~800 nm, further proving the successful loading of IR-806. Consequently, we observed more than 70-fold enhancements in Tm^3+^ emission over the whole wavelength range from 240–700 nm by Gd-CS_Y_S_2_S_3_@IR-806 compared with Gd-CS_Y_S_2_S_3_ nanoparticles, owing to the fact that the absorption cross section of Gd-CS_Y_S_2_S_3_ was significantly enhanced after IR-806 loading. Furthermore, we also observed more than 600-fold, 300-fold, 150-fold, and 30-fold enhancements in UVC (240–280 nm), UVB (280–320 nm), UVA (320–400 nm), and visible (400–700 nm) regions, respectively (Figure 2e). Similarly, we synthesized the NaGdF_4_:18%Yb,2%Er@NaYF_4_:20%Yb@NaGdF_4_:10%Yb, 50%Nd@NaGdF_4_ nanoparticles with IR-806 loading. The emission intensity in the UV spectral region increased by more than 60 times, while the intensity in the visible region increased by only 30 times (Figure S5). Taken together, these results demonstrated that the overall enhancements were dominated by increased emission in the UV spectral regions, which is consistent with the dominant effect of ligand coordination on multiphoton upconversion [37]. Notably, the enhancement factors in the UV spectral region are remarkably larger than those in the visible region, offering enticing prospects for NIR light-mediated UV upconversion nanoparticles.

### 3.3. Optimum Weight Ratio between IR-806 and Nanoparticles

**Figure 3:**
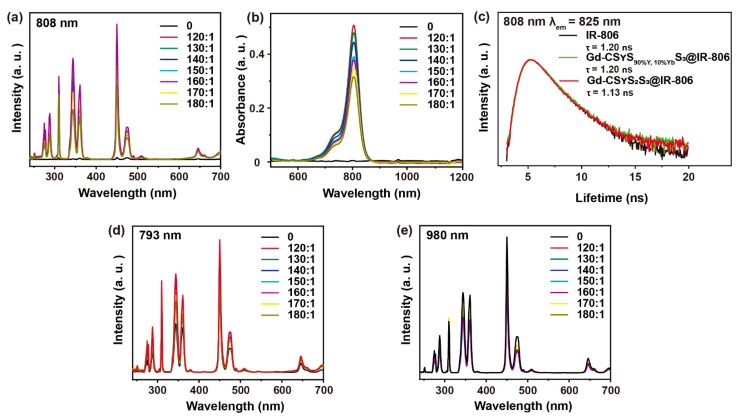
Optimizing the weight ratio of Gd-CS_Y_S_2_S_3_ to IR-806 and calculating the energy transfer efficiency. (**a**) The emission spectrum of Gd-CS_Y_S_2_S_3_ (4 mL in CHCl_3_, 0.375 mg/mL) after adding various masses of IR-806 dye under 808 nm excitation. (**b**) The absorption spectrum of Gd-CS_Y_S_2_S_3_ (4 mL in CHCl_3_, 0.375 mg/mL) with various masses IR-806 dye. (**c**) The decay curves of Gd-CS_Y_S_2_S_3_, Gd-CS_Y_S_2(90%, 10%Yb)_S_3_@IR-806, and Gd-CS_Y_S_2_S_3_@IR-806. (**d**,**e**) The emission spectra of Gd-CS_Y_S_2_S_3_ (4 mL in CHCl_3_, 0.375 mg/mL) after adding various masses of IR-806 dye under 793 nm and 980 nm excitation, respectively.

We determined the optimum weight ratio of Gd-CS_Y_S_2_S_3_:IR-806 by setting a series of weight gradients from 120:1 to 180:1 (m_NPs_:m_IR-806_). As shown in Figure 3a, the optimum weight ratio was determined to be 160:1. The optimized number of dye molecules on the surface of Gd-CS_Y_S_2_S_3_ nanoparticles was calculated to be 395 [32]. Note that the absorbance of Gd-CS_Y_S_2_S_3_@IR-806 increased as IR-806 increased. However, when the weight ratio of Gd-CS_Y_S_2_S_3_: IR-806 was smaller than 160:1, the emission intensity decreased due to fluorescence quenching caused by dye self-quenching. Due to the critical role of the Nd^3+^ sensitizers in mediating energy transfer from the dye to the upconversion nanoparticles, we verified that the optimum doping concentration of Nd^3+^ was 50 mol% (Figure S6). We then quantified the energy transfer efficiency of IR-806 to Gd-CS_Y_S_2_S_3_ by measuring the lifetime of the IR-806 in a pair of Gd-CS_Y_S_2_S_3_ samples with and without Nd^3+^ nanoparticles. Due to energy trapping by Nd^3+^, the lifetime is shortened from 1.20 ns to 1.13 ns for Gd-CS_Y_S_2_S_3_@IR-806. However, the lifetime of IR-806 was essentially unchanged after loading on Gd-CS_Y_S_90%Y,10%Yb_S_3_@IR-806, due to the absence of Nd^3+^ dopants. The energy transfer efficiency was calculated to be 5.8% according to the following equation [38]:

(1) $$E=1-\;\frac{\tau_{DA}}{\tau_{D}}$$

### 3.4. The Effect of Excitation Wavelength on UV Upconversion Emission

To investigate the enhancement effect on upconversion emission under 793, 808, and 980 nm excitation, we measured two series of Gd-CS_Y_S_2_S_3_ nanoparticles with different amounts of IR-806 loading. As shown in Figure 3d, the emission intensities of Gd-CS_Y_S_2_S_3_ were slightly improved after IR-806 loading under 793 nm excitation. In contrast, their emission intensities decreased under 980 nm excitation (Figure 3e). These results can be ascribed to poor matching between the excitation wavelengths (793 nm and 980 nm) and the absorption of IR-806. We then normalized the luminescence spectra of Gd-CS_Y_S_2_S_3_ nanoparticles under three different excitation wavelengths. We found that the ratio was unchanged for UVC, UVB, UVA, and visible spectral regions under 793 nm and 980 nm excitation. In contrast, the normalized intensity of the UVC spectral region clearly increased (Figure S7), indicating effective energy transfer from IR-806 to the nanoparticles under 808 nm excitation.

### 3.5. The Effect of IR-806 Sensitizer Distance on UV Upconversion

**Figure 4:**
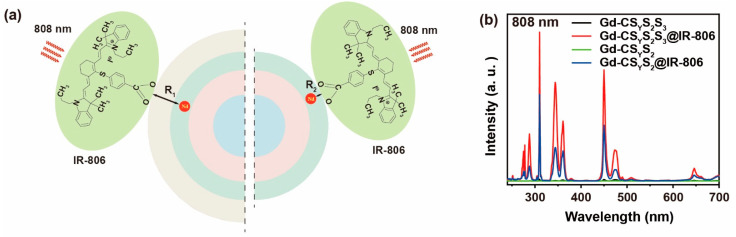
The effect of the distance between IR-806 and sensitizer Nd^3+^ on upconversion emission. (**a**) Schematic illustration of the nanostructural design to study the distance effect on upconversion emission. (**b**) The emission spectra of Gd-CS_Y_S_2_S_3_, Gd-CS_Y_S_2_S_3_@IR-806, Gd-CS_Y_S_2_, Gd-CS_Y_S_2_@IR-806 under 808 nm excitation.

To study the effect of the distance between Nd^3+^ and IR-806 on UV upconversion emission, we synthesized a pair of nanoparticles: Gd-CS_Y_S_2_S_3_ and Gd-CS_Y_S_2_ (without the third shell protection) shown in Figure 4a. Comparing the emission intensities of Gd-CS_Y_S_2_S_3_ and Gd-CS_Y_S_2_ with and without IR-806, the emission intensities of the Gd-CS_Y_S_2_ nanoparticles without shell protection increased by more than 230 times overall, while only 70-fold enhancement was observed in Gd-CS_Y_S_2_S_3_, which has 2 nm thickness shell protection. Furthermore, UV and visible emission intensities increased more than 500-fold and 130-fold, respectively, for the nanoparticles without shell protection (Figure 4b). Notably, the transfer efficiency decreased as 1/R^6^ [39]. Therefore, the enhancement factor decreased as the distance between the dye and the sensitizer increased.

Similarly, we synthesized two pairs of nanoparticles: NaGdF_4_@ NaGdF_4_:49%Yb,1%Tm@NaYF_4_:20%Yb@NaGdF_4_:10%Yb,50%Nd@NaGdF_4_ (Gd-CS_1_S_Y_S_3_S_4_) vs. NaGdF_4_@NaGdF_4_:49%Yb,1%Tm@NaYF_4_:20%Yb@NaGdF_4_:10%Yb,50%Nd (Gd-CS_1_S_Y_S_3_) and NaYF_4_@NaGdF_4_:49%Yb,1%Tm@NaYF_4_:20%Yb@NaGdF_4_:10%Yb,50%Nd@ NaGdF_4_ (Y-CS_1_S_Y_S_3_S_4_) vs. NaYF_4_@NaGdF_4_:49%Yb,1%Tm@NaYF_4_:20%Yb@NaGdF_4_:10%Yb, 50%Nd (Y-CS_1_S_Y_S_3_) (Figure S8). The core–multishell structures are illustrated in Figure S9. To study the effect of different structures on emission enhancement, NaGdF_4_ and NaYF_4_ without any dopants were used as a core to shorten the distance between the NaGdF_4_:49%Yb,1%Tm emissive layer and IR-806. The emission intensities of IR-806 grafted on Gd-CS_1_S_Y_S_3_ and Gd-CS_1_S_Y_S_3_S_4_ increased 99 and 20 times, respectively, while the luminescence intensity in the UV region increased by 118 and 25 times and that in the visible region increased by 82 and 16 times, respectively. Moreover, the emission intensities of Y-CS_1_S_Y_S_3_ and Y-CS_1_S_Y_S_3_S_4_ improved by 72 and 18 times after IR-806 loading. We also observed 81-fold and 22-fold enhancements in the UV spectral region and 63-fold and 14-fold enhancements in the visible region (Figure S10). These results are also consistent with our luminescence analysis, in that a significant enhancement in the UV luminescence of Gd-CS_Y_S_2_S_3_ nanoparticles was observed compared to the visible range (Figure S11).

### 3.6. Energy Transfer Mechanism

**Scheme 2:**
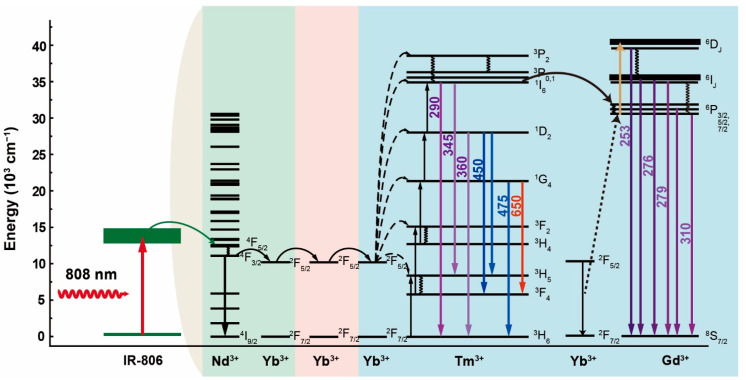
Schematic illustration of the mechanism for cascade energy transfer in Gd-CS_Y_S_2_S_3_@IR-806. Upon 808 nm laser excitation, IR-806 first absorbs excitation energy and transfers it to Nd^3+^. Next, Yb^3+^ accepts the energy from Nd^3+^, contributing to populating photons in the ^3^P_2_ state of Tm^3+^ through a continuous five-photon energy transfer process and then relaxing to the ^1^I_6_ state of Tm^3+^. Trapping the energy from both five-photon upconversion from Tm^3+^ and one-photon upconversion from Yb^3+^, six-photon and five-photon upconversion luminescence from ^6^D_J_, ^6^I_J_, and ^6^P_J_ state of Gd^3+^ is observed.

As shown in Scheme 2, IR-806 effectively absorbs the laser energy due to the absorption cross section under 808 nm excitation. To generate an efficient dye sensitization process, Nd^3+^ plays a critical role in bridging the energy transfer from the dye to the upconversion nanoparticles. Nd^3+^ ions trap the energy from the 808 nm laser and IR-806 mainly via the fluorescence–resonance energy transfer process and then gather photons at the ^4^F_5/2_ energy state. Subsequently, relaxing to the ^4^F_3/2_ energy state, Nd^3+^ transfers the energy to Yb^3+^ by an efficient energy transfer process. As an energy migrator, the excited Yb^3+^ populates the energy states of Tm^3+^ and gives rise to emission at 475 nm (^1^G_4_→^3^H_6_), 450 nm (^1^D_2_→^3^F_4_), 360 nm(^1^D_2_→^3^H_6_), 345 nm(^1^I_6_→^3^H_5_), and 290 nm(^1^I_6_→^3^H_6_). Apart from emitting, Tm^3+^ serves as an energy donor donating energy to the Gd^3+^ ions via a five-photon process. Meanwhile, the six-photon upconversion process of 253 nm (^6^D_9/2_→^8^S_7/2_) and the five-photon upconversion processes of 273 nm (^6^I_J_→^8^S_7/2_), 276 nm (^6^I_J_→^8^S_7/2_), 279 nm (^6^I_J_→^8^S_7/2_), 306 nm (^6^P_5/2_→^8^S_7/2_), and 310 nm (^6^P_7/2_→^8^S_7/2_) are observed with the assistance of the appropriate energy matching of the following transition of ^2^F_5/2_→^2^F_7/2_ (9750 cm^−1^, Yb^3+^): ^6^P_J_→^6^D_J_ (∼8750 cm^−1^, Gd^3+^). Notably, the utilization of an optically inert NaYF_4_ host lattice with Yb^3+^ dopants as the interlayer plays a decisive role in protecting the energy by cooperative dye and Nd^3+^ sensitization from interior lattice defects, making it possible to effectively further increase UV via dye sensitizing.

### 3.7. Back Energy Transfer from Nanoparticles to IR-806

**Figure 5:**
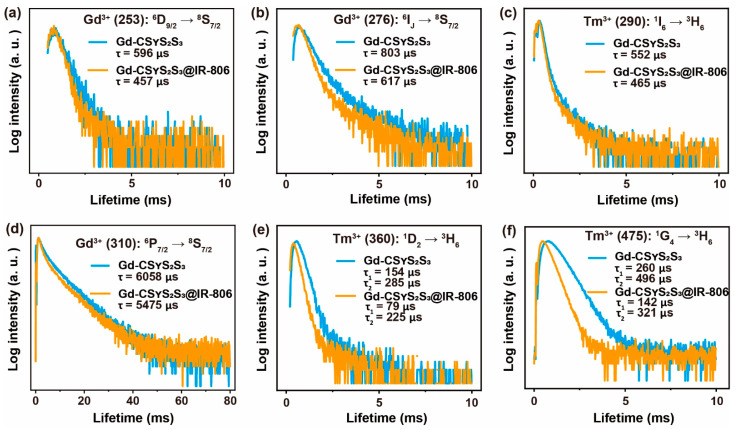
The decreased lifetime of Tm^3+^ and Gd^3+^ for Gd-CS_Y_S_2_S_3_@IR-806. (**a**–**f**) The Tm^3+^ and Gd^3+^ lifetime decay curves of Gd-CS_Y_S_2_S_3_ and Gd-CS_Y_S_2_S_3_@IR-806 at 253, 276, 290, 310, 360, and 475 nm under 808 nm excitation, respectively.

As well as increasing the luminescence intensity, a back energy transfer process from IR-806 to Gd-CS_Y_S_2_S_3_ occurred. As depicted in Figure 5 and Figure S12, the lifetime of Gd^3+^ at 253, 276, and 310 nm, and Tm^3+^ at 290, 345, 475, and 650 nm slightly decreased after IR-806 loading, which can be ascribed to the nonradiative energy transfer from Gd^3+^ and Tm^3+^ to IR-806 [40,41,42].

## 4. Discussion

In this study, we developed a dye-sensitized heterogeneous lanthanide nanoparticle to regulate the energy transfer pathway for UV enhancement by 808 nm excitation. We systematically studied the influence of dye concentration, excitation wavelength, and distance between the dye and the sensitizer Nd^3+^ on upconversion emission, especially in the UV spectral region. Dye loading can improve the absorption of excitation light and thus improve the efficiency of energy-transfer-mediated upconversion. Moreover, our experimental results demonstrated a strengthened multiphoton upconversion process, which can be ascribed to the dominant effect of ligand loading on upconversion emission from high-lying energy states. The fundamentals gained from our investigations may provide insights into promoting the multiphoton upconversion process and the future design of organic–inorganic hybrid luminescent nanoparticles for applications in photocatalysis, biomedicine, environmental science, and more.

## Data Availability

All of the relevant data are available from the correspondence authors upon reasonable request. Source data are provided with this paper.

## Supplementary Materials

The following are available online at https://www.mdpi.com/article/10.3390/nano11113114/s1. Figure S1: TEM and size distribution of Gd-CSYS2S3 nanoparticles; Figure S2: XRD of Gd-CSYS2S3 nanoparticles; Figure S3: EDX of Gd-CSYS2S3 nanoparticles; Figure S4: EDX lining analysis of Gd-CSYS2S3 nanoparticles; Figure S5: luminescence emission of NaGdF4:18%Yb,2%Er@NaYF4:20%Yb@NaGdF4:10%Yb, 50%Nd@NaGdF4 with and without IR-806 loading; Figure S6: luminescence emission of Gd-CSYS2S3 nanoparticles with different Nd3+ doping before and after IR-806 loading; Figure S7: normalized intensity of luminescence spectra of Gd-CSYS2S3 with various contents of IR-806; Figure S8: TEM images of as-synthesized nanoparticles with different structures for distance effect studies; Figure S9: schematic illustration of five types of core–multishell structures including Gd-CSYS2S3, Y-CS1SYS3, Y-CS1SYS3S4, Gd-CS1SYS3, and Gd-CS1SYS3S4; Figure S10: luminescence spectra of as-synthesized nanoparticles with different structures for distance effect studies; Figure S11: normalized intensities of luminescence spectra of corresponding nanoparticles for distance effect studies; Figure S12: the lifetime decay of Tm3+ at 650 nm in Gd-CSYS2S3 and Gd-CSYS2S3@IR-806 nanoparticles under 808 nm excitation.
