# Supplementary material for: Dye Sensitization for Ultraviolet Upconversion Enhancement
Source: Nanomaterials (Basel). 2021 Nov 18;11(11):3114. doi: 10.3390/nano11113114 (PMC8623389; doi:10.3390/nano11113114)
Supplement: Supplementary file 1 [file nanomaterials-11-03114-s001.zip › nanomaterials-1449237-supplementary.pdf]

Supplementary Materials

Dye Sensitization for Ultraviolet Upconversion Enhancement

Ming–kai Wang [1], Han-Lin Wei [1], Shuai Wang [1], Chuanyu Hu [2,3,4,*], Qianqian Su [1,*]

[1] Institute of Nanochemistry and Nanobiology, Shanghai University, Shanghai 200444, P. R. China
[2] Department of Stomatology, Tongji Hospital, Tongji Medical College, Huazhong University of Science and Technology, Wuhan 430030, China
[3] School of Stomatology, Tongji Medical College, Huazhong University of Science and Technology, Wuhan 430030, China
[4] Hubei Province Key Laboratory of Oral and Maxillofacial Development and Regeneration, Wuhan 430022, China
* Correspondence: chmsqq@shu.edu.cn (Q. S.); chuanyuhu@hust.edu.cn (C. H.)

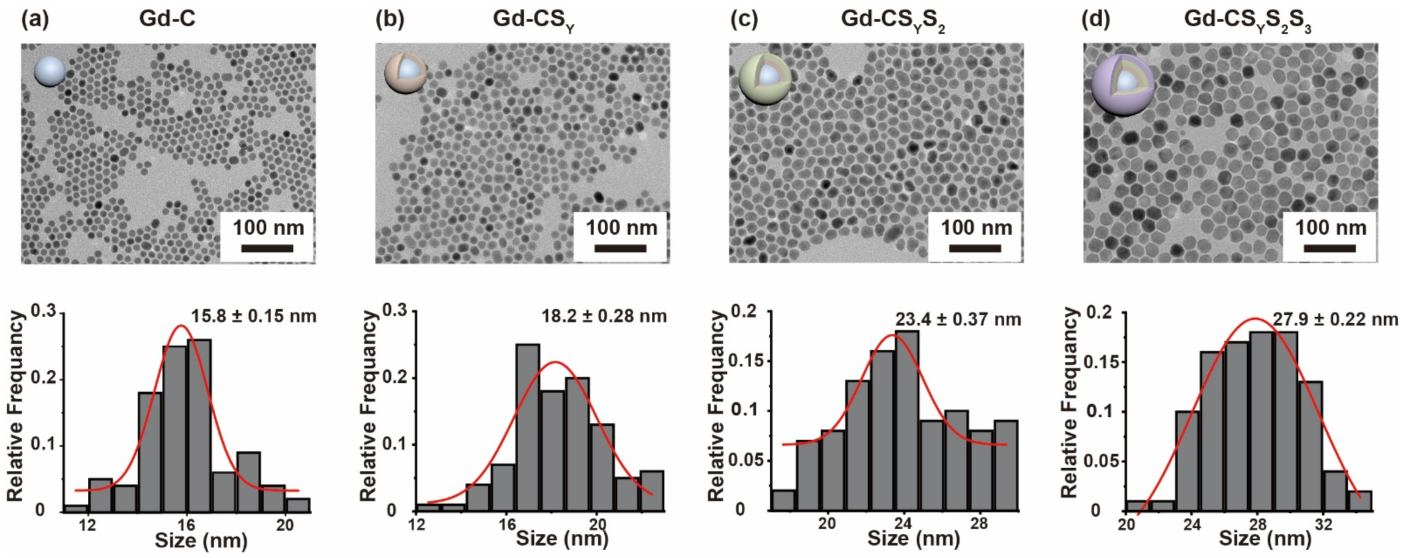

Figure S1. Transmission electron microscopy (TEM) and nanoparticles size distribution of as-prepared $NaGdF_4$:49%Yb,1%Tm@$NaYF_4$:20%Yb@$NaGdF_4$:10%Yb,50%Nd@$NaGdF_4$ (Gd-$CS_YS_2S_3$). (a) core, (b) core-shell, (c) core-shell-shell, (d) core-multishell. The size distribution of nanoparticles is matched by a Gaussian curve.

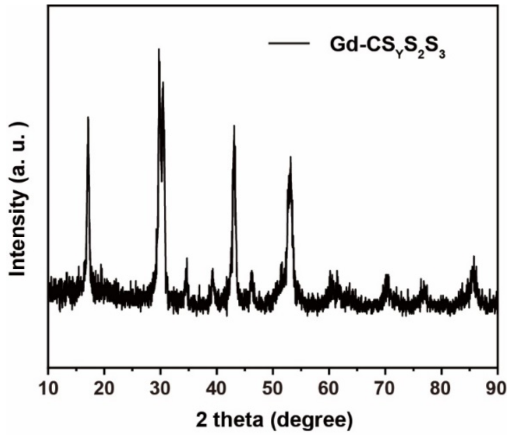

Figure S2. X-ray powder diffraction (XRD) curve of the as-synthesized heterogeneous core-multishell nanoparticles.

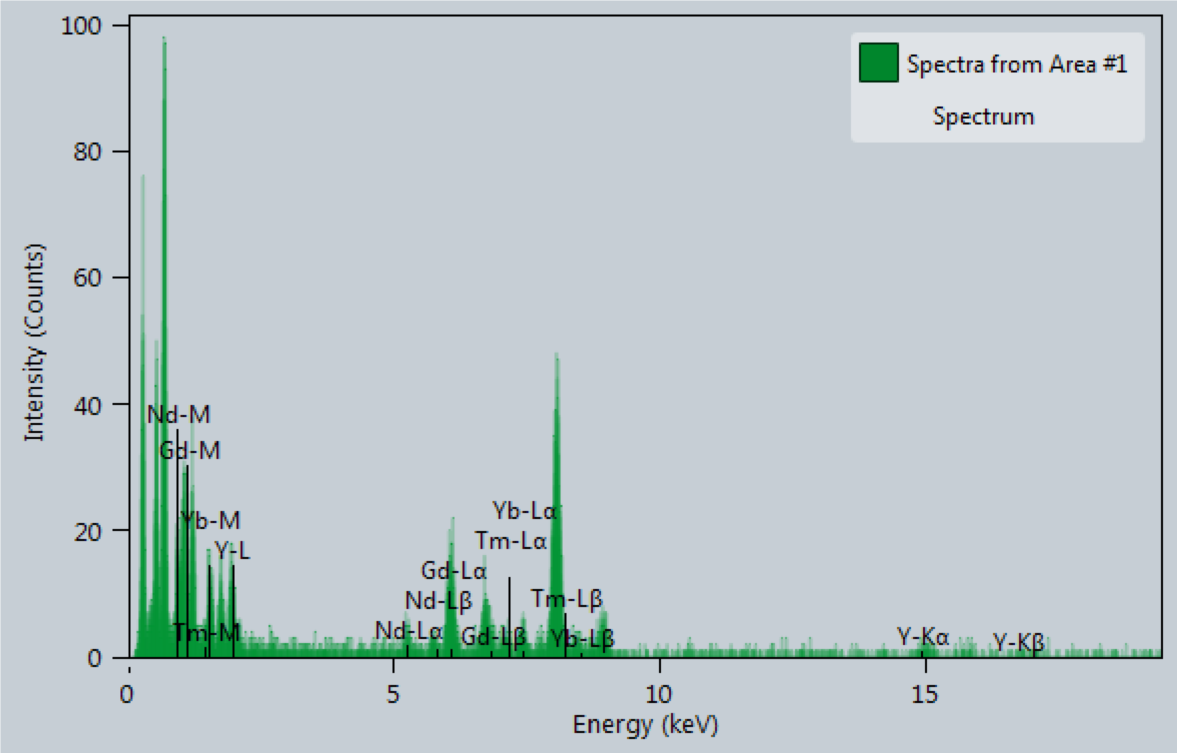

Figure S3. Energy dispersive X-ray (EDX) spectrum of Gd-$CS_YS_2S_3$ nanoparticles.

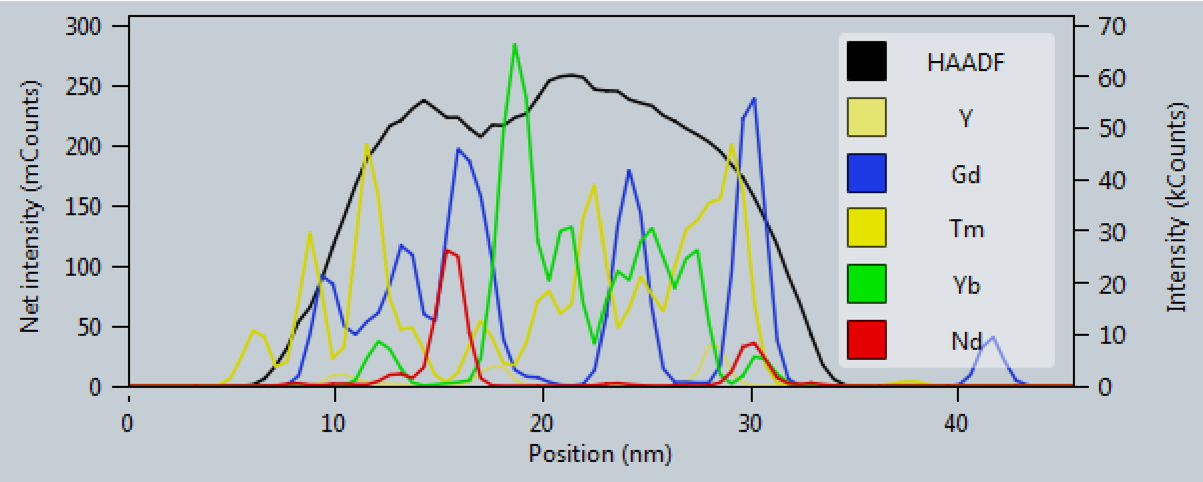

Figure S4. Energy-dispersive X-ray lining analysis of the as-synthesized heterogeneous core-multishell nanoparticles.

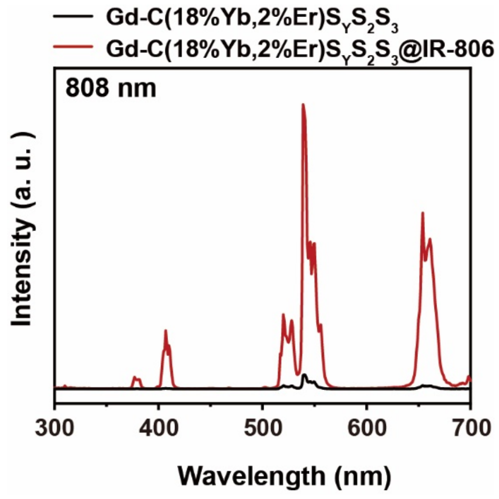

Figure S5. The luminescence emission spectrum of $NaGdF_4$:18%Yb,2%Er@$NaYF_4$:20%Yb@$NaGdF_4$: 10%Yb,50%Nd@$NaGdF_4$(Gd-C(18%Yb,2%Er)$S_YS_2S_3$) with and without IR-806 under 808 nm laser excitation.

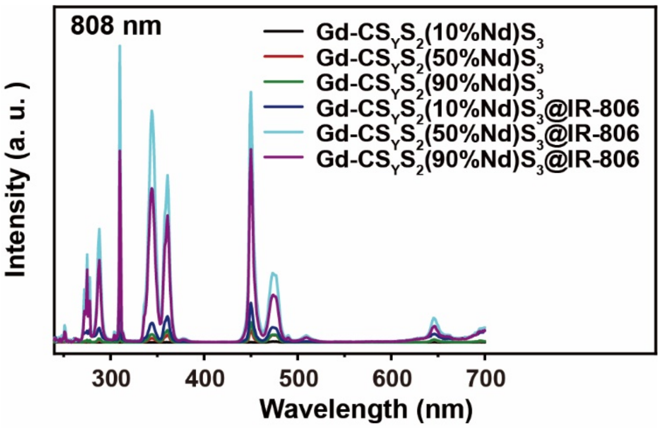

Figure S6. The luminescence emission spectrum of $NaGdF_4$:49%Yb,1%Tm@$NaYF_4$:20%Yb@ $NaGdF_4$:x%Nd (x = 10%, 50%, 90%), 10%Yb@$NaGdF_4$ with and without IR-806 for 808 nm laser exciting.

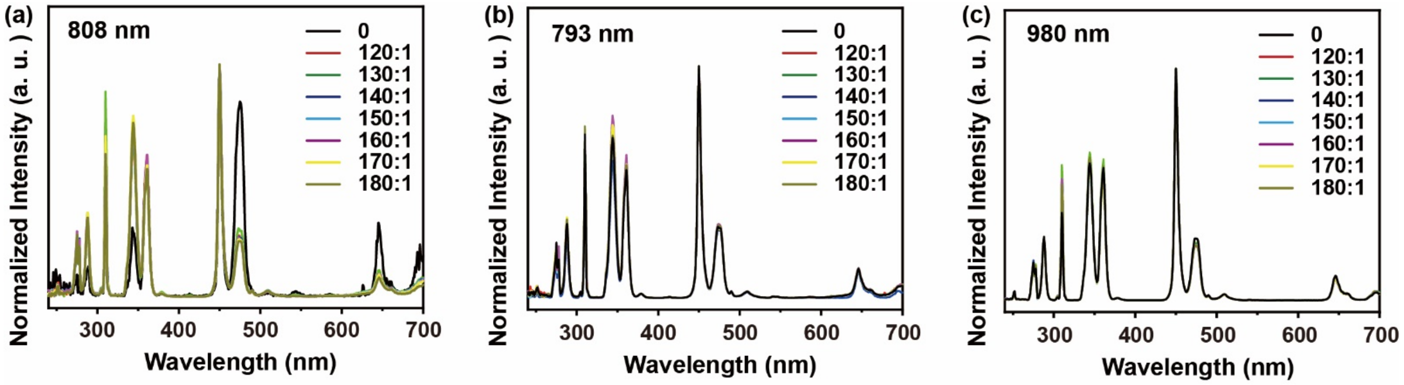

Figure S7. Normalized intensity of luminescence spectra of Gd-$CS_YS_2S_3$ with various content of IR-806 (m_{NP} : m_{IR-806} = 0, 120:1, 130:1, 140:1, 150:1, 160:1, 170:1, 180:1) under 808 nm, 793 nm and 980 nm.

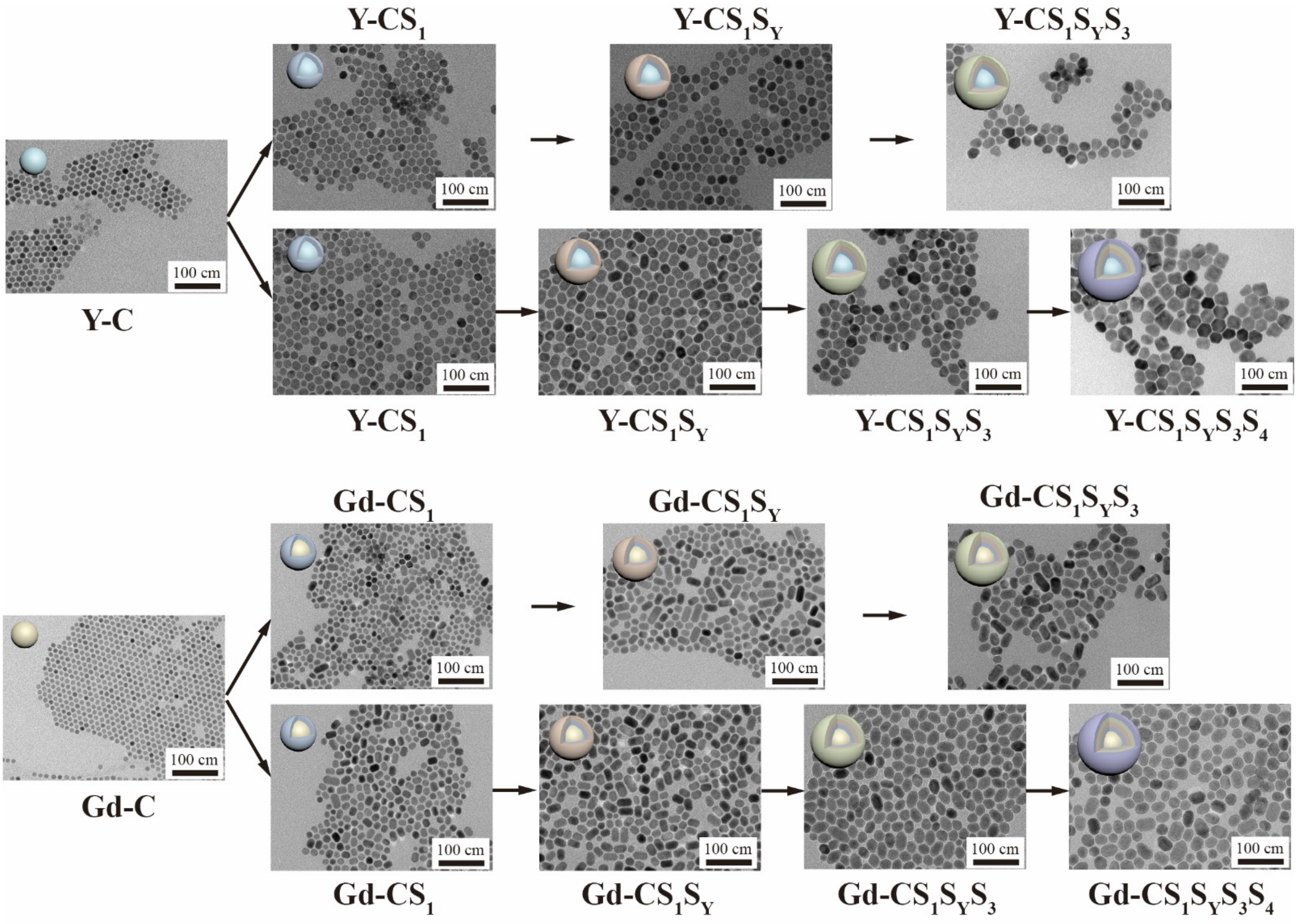

Figure S8. Transmission electron microscopy (TEM) and particles size distribution of as-prepared $NaYF_4$@$NaGdF_4$:49%Yb,1%Tm@$NaYF_4$:20%Yb@$NaGdF_4$:10%Yb,50%Nd (Y-$CS_1S_YS_3$), $NaYF_4$@$NaGdF_4$: 49%Yb,1%Tm@$NaYF_4$:20%Yb@$NaGdF_4$:10%Yb,50%Nd@$NaGdF_4$ (Y-$CS_1S_YS_3S_4$), $NaGdF_4$@$NaGdF_4$: 49%Yb,1%Tm@$NaYF_4$:20%Yb@$NaGdF_4$:10%Yb,50%Nd (Gd-$CS_1S_YS_3$), $NaGdF_4$@$NaGdF_4$:49%Yb,1%Tm @$NaYF_4$:20%Yb@$NaGdF_4$:10%Yb,50%Nd@$NaGdF_4$ (Gd-$CS_1S_YS_3S_4$).

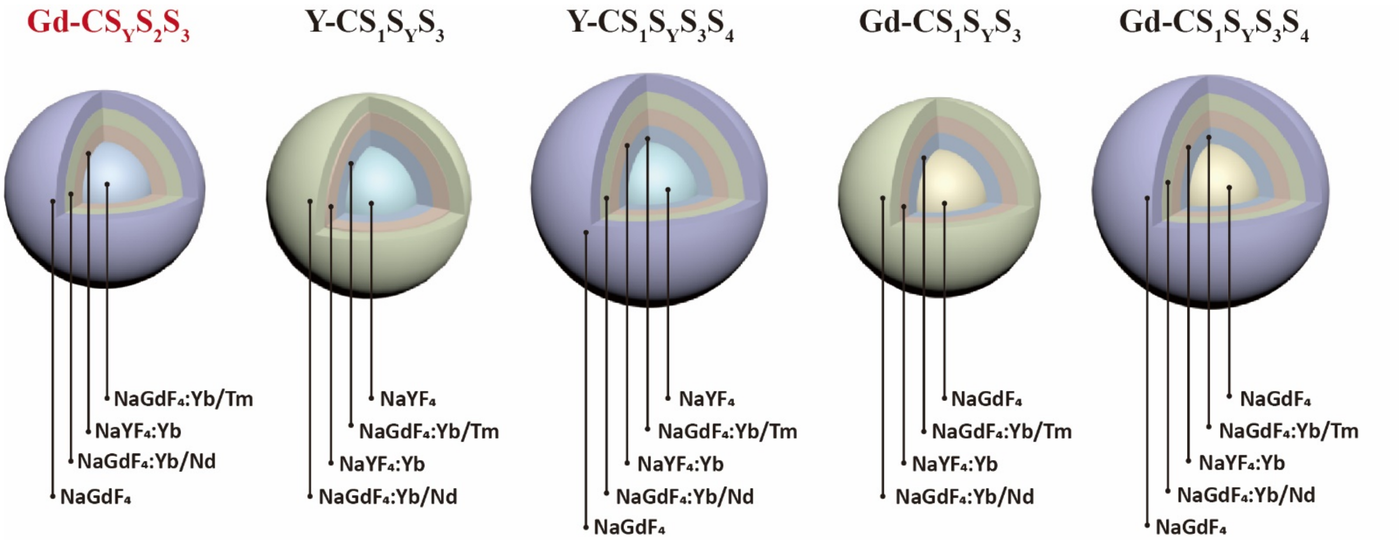

Figure S9. Schemestic illustration of five types core-multishell structures including $NaGdF_4$: 49%Yb,1%Tm@$NaYF_4$:20%Yb@$NaGdF_4$:10%Yb,50%Nd@$NaGdF_4$ (Gd-CS$_Y$S$_2$S$_3$), $NaYF_4$@$NaGdF_4$:49%Yb,1%Tm@$NaYF_4$:20%Yb@$NaGdF_4$:10%Yb,50%Nd (Y-CS$_1$S$_Y$S$_3$), $NaYF_4$@$NaGdF_4$: 49%Yb,1%Tm@$NaYF_4$:20%Yb@$NaGdF_4$:10%Yb,50%Nd@$NaGdF_4$ (Y-CS$_1$S$_Y$S$_3$S$_4$), $NaGdF_4$@$NaGdF_4$: 49%Yb,1%Tm@$NaYF_4$:20%Yb@$NaGdF_4$:10%Yb,50%Nd (Gd-CS$_1$S$_Y$S$_3$), $NaGdF_4$@$NaGdF_4$:49%Yb,1%Tm @$NaYF_4$:20%Yb@$NaGdF_4$:10%Yb,50%Nd@$NaGdF_4$ (Gd-CS$_1$S$_Y$S$_3$S$_4$).

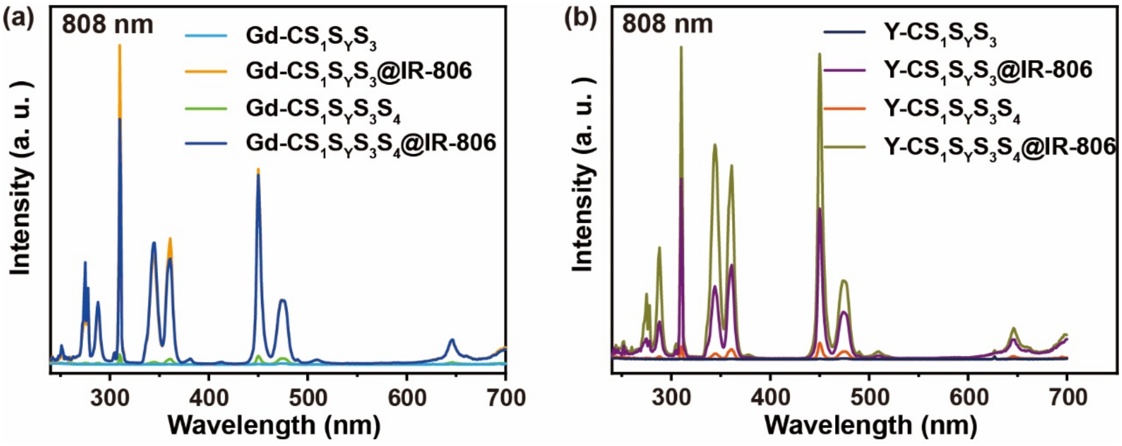

Figure S10. Luminescence emission spectra of Gd-CS$_1$S$_Y$S$_3$, Gd-CS$_1$S$_Y$S$_3$@IR-806, Gd-CS$_1$S$_Y$S$_3$S$_4$, Gd-CS$_1$S$_Y$S$_3$S$_4$@IR-806 and Y-CS$_1$S$_Y$S$_3$, Y-CS$_1$S$_Y$S$_3$@IR-806, Y-CS$_1$S$_Y$S$_3$S$_4$, Y-CS$_1$S$_Y$S$_3$S$_4$@IR-806 under 808 nm CW diode lasering, with excitation power is 10 W/cm^2.

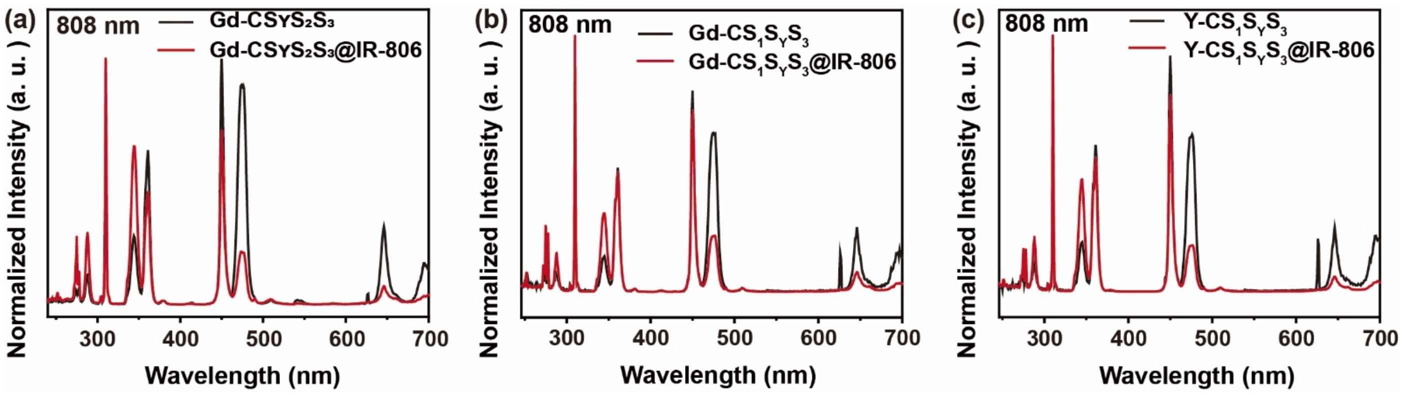

Figure S11. Normalized intensity of luminescence spectra of Gd-CS$_Y$S$_2$S$_3$ and Gd-CS$_Y$S$_2$S$_3$@IR-806, Gd-CS$_1$S$_Y$S$_3$ and Gd-CS$_1$S$_Y$S$_3$@IR-806, Y-CS$_1$S$_Y$S$_3$ and Y-CS$_1$S$_Y$S$_3$@IR-806 by 808 nm lasering.

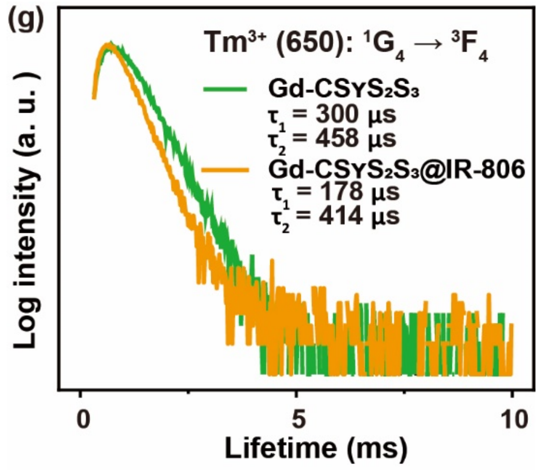

Figure S12. The decay curves of Tm^{3+} at 650 nm in Gd-CS$_Y$S$_2$S$_3$ and Gd-CS$_Y$S$_2$S$_3$@IR-806 nanoparticles under 808 nm excitation.
